# Optical neuroimaging: advancing transcranial magnetic stimulation treatments of psychiatric disorders

**DOI:** 10.1186/s42492-022-00119-y

**Published:** 2022-09-08

**Authors:** Shixie Jiang, Linda L. Carpenter, Huabei Jiang

**Affiliations:** 1 Department of Psychiatry and Behavioral Sciences, Stanford University School of Medicine, Palo Alto, CA 94305 USA; 2 Department of Psychiatry and Human Behavior, Butler Hospital, Alpert Medical School of Brown University, Providence, RI 02903 USA; 3 Department of Medical Engineering, University of South Florida, Tampa, FL 33613 USA

**Keywords:** Optical imaging, Functional near-infrared spectroscopy, Diffuse optical tomography, Transcranial magnetic stimulation, Major depressive disorder, Panic disorder, Phobia, Bulimia nervosa, Psychiatric disorders

## Abstract

Transcranial magnetic stimulation (TMS) has been established as an important and effective treatment for various psychiatric disorders. However, its effectiveness has likely been limited due to the dearth of neuronavigational tools for targeting purposes, unclear ideal stimulation parameters, and a lack of knowledge regarding the physiological response of the brain to TMS in each psychiatric condition. Modern optical imaging modalities, such as functional near-infrared spectroscopy and diffuse optical tomography, are promising tools for the study of TMS optimization and functional targeting in psychiatric disorders. They possess a unique combination of high spatial and temporal resolutions, portability, real-time capability, and relatively low costs. In this mini-review, we discuss the advent of optical imaging techniques and their innovative use in several psychiatric conditions including depression, panic disorder, phobias, and eating disorders. With further investment and research in the development of these optical imaging approaches, their potential will be paramount for the advancement of TMS treatment protocols in psychiatry.

## Introduction

Transcranial magnetic stimulation (TMS) was first developed in 1985 by Barker et al. [1]. Since its conception, it has rapidly become an important research and clinical tool for the study and treatment of various psychiatric disorders [2]. The alternating magnetic field created by passing an electrical current through an insulated coil is capable of noninvasively modulating neural activity. This magnetic field penetrates the scalp and skull directly beneath the coil to induce neuronal depolarization and subsequent activation or inhibition [3]. In principle, TMS can therefore be used to promote improvement of abnormal neural activity and restore the dysfunctional brain networks that underpin psychiatric symptoms. Over the past decade, TMS has become a standard therapy for adults with depression who are resistant to traditional treatments, and numerous clinical trials have shown the benefits of TMS for a wide array of neuropsychiatric conditions. However, its effectiveness has likely been limited due to the dearth of neuronavigational tools for targeting purposes, unclear ideal stimulation parameters, and a lack of knowledge regarding the physiological response of the brain to TMS in each psychiatric condition [4]. As such, the development of neuroimaging modalities has been a promising area of research that has been pursued in order to understand the mechanisms of TMS and improve its overall impact.

Unlike strokes or other focal neurological disorders, psychiatric disorders typically do not cause visible changes on standard structural imaging techniques, including magnetic resonance imaging (MRI) and computed tomography (CT) [5]. Instead, disorders such as depression or schizophrenia can only be imaged using functional imaging modalities that depict the activity of abnormal brain networks [6]. These techniques include functional magnetic resonance imaging (fMRI), single photon emission computed tomography (SPECT), positron emission tomography (PET), magnetoencephalography (MEG), and electroencephalography (EEG) [7–9]. Neuroimaging has thus offered valuable insights into the pathophysiology of various psychiatric disorders in recent decades and has been crucial for identifying promising biomarkers and treatment targets. However, despite the usefulness of these modalities, their application can be limited in the pursuit of improving the critical TMS parameters, as described above.

Several studies have utilized fMRI, SPECT, and EEG to predict outcomes and optimize TMS treatment approaches for psychiatric disorders [10–12]. One of the most promising findings to date comes from the development of connectivity analysis software using resting-state fMRI data to guide TMS coil placement to an optimal scalp location over the dorsolateral prefrontal cortex (DLPFC) for more precise targeting and improved outcomes in depression [13]. However, none of these imaging modalities can be used concurrently with TMS to provide information about dynamic brain changes during active stimulation sessions. Without knowledge of how the brain responds during a stimulation interval, personalization and optimization of spatial targets, pulse frequencies, treatment intensity, and other aspects of TMS delivery cannot be achieved. While fMRI-based targeting holds considerable potential, the acquisition of MRI data is not safe or feasible for many psychiatric patients because of the presence of non-removable metal or the inability to fit or remain still for prolonged periods in the scanner. Importantly, EEG, fMRI, and MEG are the only modalities that possess the temporal resolution necessary to evaluate any acute response to brain stimulation [14–16]. Unfortunately, they all rely on signals that involve the electromagnetic spectrum, and TMS produces very potent magnetic and electrical fields. Thus, the overall resolution and quality of the images and data generated by these imaging techniques are subject to significant measurement artifacts. To understand the acute physiological neural responses produced by TMS and targeted stimulation to modulate a specific brain region or network, no technique is adequate. One solution to this conundrum has emerged from the advent and development of optical imaging techniques that can be used in humans.

### Optical neuroimaging

In 1977, Jöbsis [17] was the first to demonstrate the feasibility of measuring blood and tissue oxygenation changes in the brain of a living organism by employing near-infrared (NIR) light. Since then, the field of optical neuroimaging has progressed into a robust and diverse area of research with many basic science, translational, and clinical studies that have benefited from the unique elements of light to image the brain. NIR light can provide important functional imaging data by detecting intrinsic changes in absorption, fluorescence, or scattering. A broad armamentarium of exogenous contrast media can be utilized as well to capture further data [18]. Oxy- and de-oxyhemoglobin are chromophores that, along with cytochromes and metabolites, are commonly measured to capture markers of functional activation within the brain, similar to conventional functional imaging techniques that estimate activity [19]. Optical imaging provides a plethora of benefits though over standard functional imaging modalities. These include real-time capabilities, reduced costs, decreased subject movement restriction, a wider variety of contrast agents, lack of ionizing radiation, and portability [18]. Additionally, for the purpose of studying the effects of TMS on the brain, optical imaging does not cause any significant electromagnetic interferences due to inherent property differences. Thus, concurrent imaging of the acute modulation of brain activity during a TMS session can be accomplished using NIR-based technology. To study the effects of TMS in patients with psychiatric disorders, two notable optical imaging techniques have been successfully utilized: functional near-infrared spectroscopy (fNIRS) and diffuse optical tomography (DOT).

fNIRS is based on the basic principles of NIR spectroscopy, and the term, ‘functional’ was denoted after its use in the first human studies published in the early 1990s [20, 21]. fNIRS is capable of detecting changes in the optical properties of the human cortex from multiple measurement sites simultaneously, with the results displayed in a topographical map or image over a precise region. It exploits the fact that human tissues are transparent to light in the NIR spectral window (650–1000 nm) and NIR light is effectively absorbed by chromophores (e.g., hemoglobin in small vessels) or scattered in tissues [22]. By measuring increases in oxy-hemoglobin and concomitant decreases in de-oxyhemoglobin, fNIRS captures acute markers of increased local arteriolar vasodilation, thus increasing local cerebral blood flow and volume in targeted areas. This hemodynamic response highlights the concept of neurovascular coupling, which is then used as a surrogate for neuronal activity in the brain [23]. The benefits of fNIRS for investigating TMS effects include the aforementioned reasons, with an emphasis on portability, low cost, and compatibility with concurrent stimulation. There are multiple companies marketing multichanneled fNIRS devices that can be purchased for a fraction of the price of an fMRI scanner. Given the small size of the interface and the computer setup [24], these devices can be easily operated and readily moved from subject to subject. DOT is a modern noninvasive NIR-based technique that can be viewed as an extension of fNIRS, similar to the distinction between magnetic resonance spectroscopy and MRI. Unlike the two-dimensional topographic approach of fNIRS, DOT uses multiple NIR wavelengths and overlapping channels, along with a range of source-detector distances for data acquisition [25]. This allows for the quantification of hemodynamic responses occurring at various depths within the brain and the production of three-dimensional images of high spatial and temporal resolution [26]. By obtaining volumetric functional information, DOT offers an attractive alternative approach to three-dimensional functional neuroimaging. Although both of these modalities focus on blood oxygen level-dependent signals, the difference between them lies in the dependence of fMRI on the measurement of mainly de-oxyhemoglobin [27]. Moreover, since DOT can simultaneously measure all types of hemoglobin signals, this creates the capability to distinguish differences in the timing, localization, and magnitude of neurovascular coupling that escape detection by fMRI, thus generating a more informative image of dynamic changes in the brain [28–31]. Notably, similar to fNIRS, DOT is also quite inexpensive, portable, and mobile, and there are new systems of wearable DOT devices with increasingly flexible interfaces on the horizon [32–34].

### Concurrent optical neuroimaging and TMS in psychiatric disorders

In psychiatric disorders, fNIRS and DOT have been successfully utilized for the concurrent imaging of the brain during TMS. Studies have focused on patients with various diagnoses, including depressive, anxiety, and eating disorders. The primary advantage of these techniques is that both may offer useful metrics for evaluating treatment response, disease status, symptom severity, or functional targeting for the optimization of TMS treatment parameters. Furthermore, they provide an attractive option to study and understand the neurophysiological effects of TMS. Table 1 provides a summary of published studies using fNIRS or DOT in relation to the application of TMS.

**Table 1:** Summary of published studies using optical neuroimaging concurrently with TMS

Reference	Psychiatric disorder	Sample size	Optical imaging technique	Imaging site (s)	Activation task	TMS stimulation parameters	Stimulation site	Results
Eschweiler et al., 2000 [35]	Major depressive disorder	12	fNIRS	Bilateral prefrontal cortex	Mental arithmetic; left and right-handed mirror drawing	10 Hz, 10 s duration, 20 trains total, 90% RMT	Left DLPFC	Pre-TMS [HbT] changes during task correlated with changes in HAM-D scores
Shinba et al., 2018 [36]	Major depressive disorder	15	fNIRS	Bilateral frontal cortex	None; resting state	10 Hz, 4 s duration, 26 s inter-train interval, 3000 pulses, 120% RMT	Left DLPFC	Maintenance and magnitude of [HbO] during stimulation session was related to effectiveness of TMS
Huang et al., 2022 [37]	Major depressive disorder	40 depressed; 40 healthy	fNIRS	Bilateral prefrontal cortex	Verbal fluency task	2 Hz, 10 s duration, 3 s inter-train interval, 2600 pulses, 90% RMT	Right DLPFC	Increased [HbO] from baseline to post-treatment in depressed patients positively correlated to reduction of HAM-D scores
Jiang et al., 2021 [38]	Major depressive disorder	7 depressed; 6 healthy	DOT	Contralateral DLPFC	None; resting state	10 Hz, 4 s duration, 26 s inter-train interval, 3000 pulses,100% RMT	Left DLPFC	Depressed subjects observed to have a lower magnitude and volume of [HbO] and [HbT]
Dresler et al., 2009 [39]	Panic disorder	1	fNIRS	Bilateral prefrontal cortex	Emotional Stroop task	10 Hz, 2 s stimulation, 8 s inter-train interval, 2400 pulses, 120% RMT	Left DLPFC	Increased bilateral [HbO] after TMS treatment
Deppermann et al., 2014 [40]	Panic disorder	44	fNIRS	Bilateral prefrontal cortex	Verbal fluency task	15 pulses per second, 2 s duration, 600 pulses, 80% RMT	Left DLPFC	Increase in [HbO] in left inferior frontal gyrus during and post-TMS correlated with anxiety scores
Deppermann et al., 2017 [41]	Panic disorder	44	fNIRS	Bilateral prefrontal cortex	Emotional Stroop task	15 pulses per second, 2 s duration, 600 pulses, 80% RMT	Left DLPFC	Increased [HbO] in bilateral regions during and post-TMS
Deppermann et al., 2016 [42]	Phobia	41 with phobia; 42 healthy	fNIRS	Bilateral prefrontal cortex	Emotional Stroop task	15 pulses per second, 2 s duration, 600 pulses, 80% RMT	Left DLPFC	Increased [HbO] in bilateral regions during and post-TMS
Sutoh et al., 2016 [43]	Bulimia nervosa	8	fNIRS	Bilateral prefrontal cortex	Food photo task	10 Hz, 5 s duration, 55 s inter-train interval, 1000 pulses, 110% RMT	Left DLPFC	Significant decrease in [HbO] in left DLPFC after single treatment

*RMT* Resting motor threshold, *HbT* Hemoglobin concentration, *HbO* Oxyhemoglobin concentration, *HAM-D* Hamilton Depression Rating Scale

In 2000, Eschweiler et al. [35] were the first to demonstrate the potential utility of optical neuroimaging for informing TMS therapy. In a small sham-controlled cross-over study for patients with major depressive disorder, fNIRS was employed to measure hemoglobin changes in the prefrontal cortex during a computer-based task (mental arithmetic or left or right-handed mirror drawing). The absence of a task-related increase in total hemoglobin concentration predicted the clinical response to a course of active repetitive TMS (rTMS) in the trial. Interestingly, the paper describing these initial results was published during the ascent of fMRI studies designed to understand the neural circuitry underlying depression, and prior to any fMRI-TMS paired imaging studies [44]. The dominance of fMRI in the field of functional imaging may be the reason why, despite these positive results, no follow-up investigation was attempted after the Eschweiler study until 2018, when Shinba et al. [36] published their findings. In a cohort of patients with treatment refractory depression, fNIRs was used to continuously record hemoglobin changes over the bilateral frontal cortex during two entire rTMS sessions (37.5 mins per session, first and last treatments in the series). Their results demonstrated that higher values of the frontal lobe hemodynamic response during stimulation correlated with greater reductions in depressive symptoms as a patient approached the end of the TMS treatment course. Huang et al. [37] further validated the fNIRS-TMS approach in a larger study in which fNIRS measurements during a verbal fluency task were used to predict treatment response to TMS and to monitor normalization of hemodynamic response to the task.

**Fig. 1:**
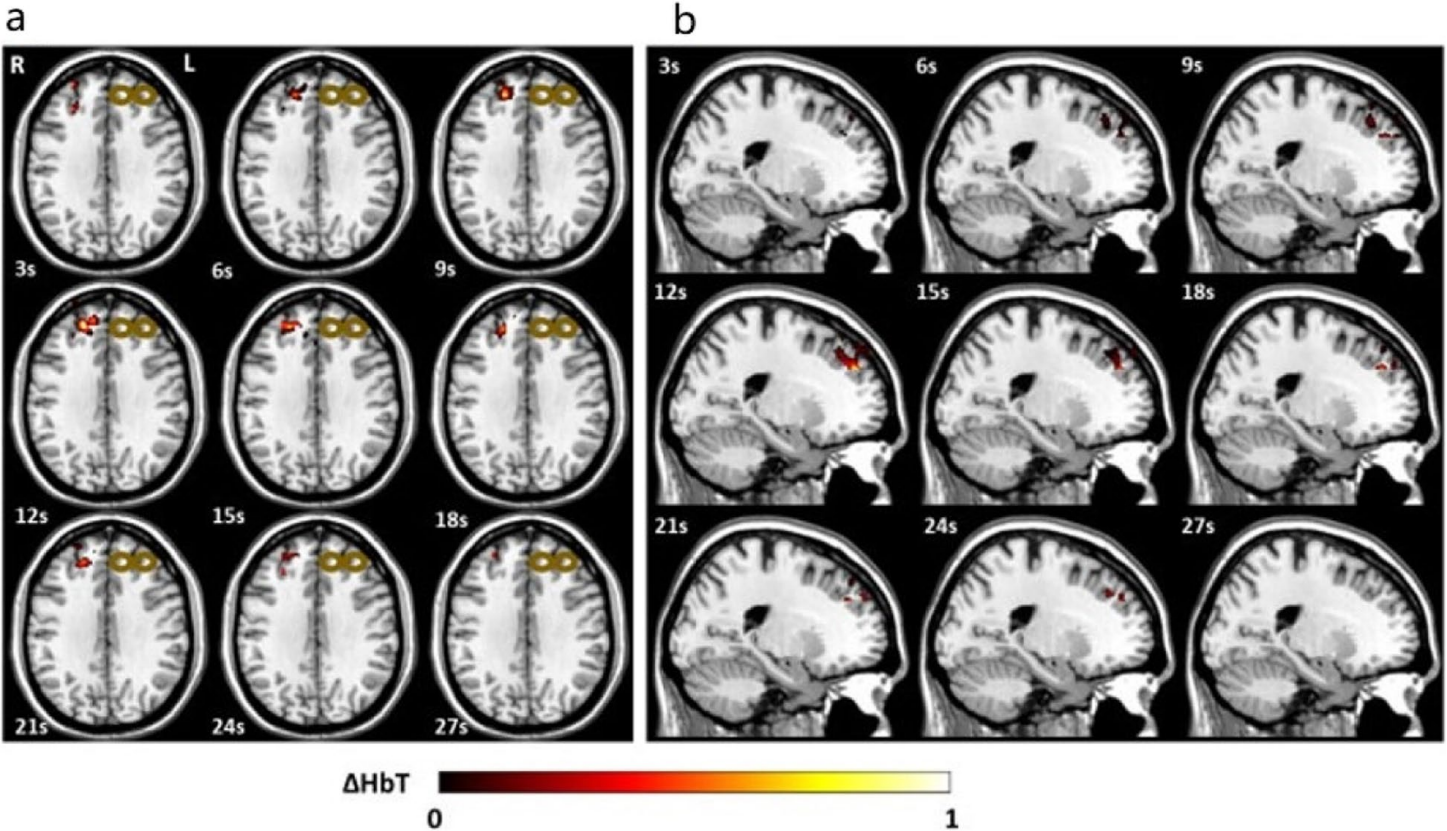
**a** Transverse view of the three-dimensional [HbT] images collected by DOT in a healthy subject during a 30-s epoch of TMS; **b** Sagittal view of the three-dimensional [HbT] images collected by DOT in a healthy subject during a 30-s epoch of TMS. The bronze colored coil symbol represents stimulation of the left dorsolateral prefrontal cortex [38]

**Fig. 2:**
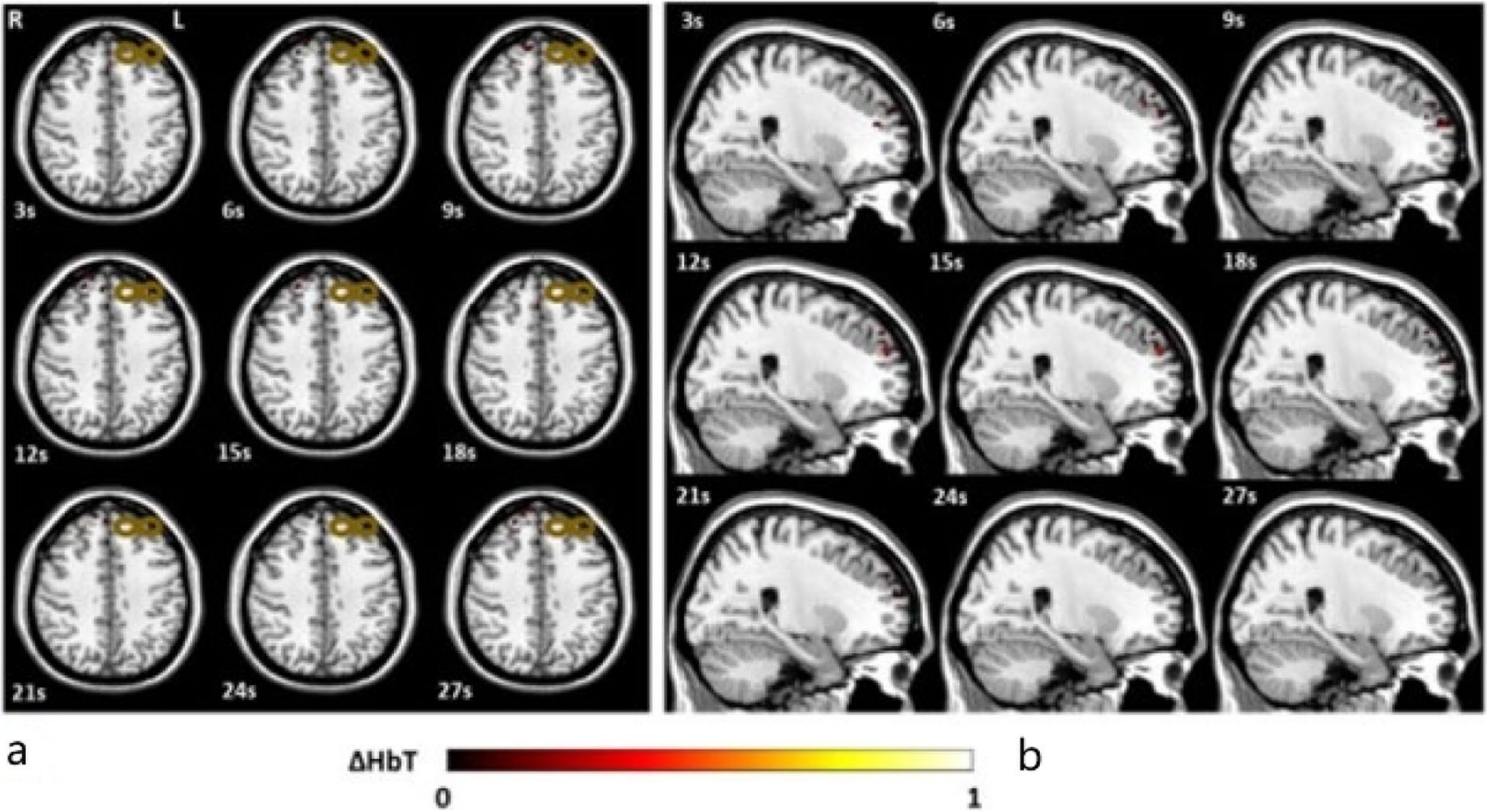
**a** Transverse view of the three-dimensional [HbT] images collected by DOT in a depressed subject during a 30-s epoch of TMS; **b** Sagittal view of the three-dimensional [HbT] images collected by DOT in a depressed subject during a 30-s epoch of TMS. The bronze colored coil symbol represents stimulation of the left dorsolateral prefrontal cortex [38]

In 2021, our group applied DOT imaging concurrent with stimulation for the first time to demonstrate that depressed patients, in comparison with healthy subjects, had abnormal neurovascular responses to TMS, as evidenced by a decreased volume and magnitude of DLPFC activation during TMS treatments (Figs. 1 and 2) [38]. TMS-evoked hemodynamics were immediately blunted in depressed patients and remained diminished during the entire stimulation and inter-training periods of a treatment session. In contrast, healthy subjects had much more robust and enhanced responses, reflecting the recruitment of greater DLPFC volume. While previous experiments with fNIRS have focused only on the two-dimensional response to TMS, the inclusion of depth information with DOT may provide a precise approach for biomarker development to guide TMS parameters and optimize brain targeting.

Offline assessments of cortical hemodynamics during cognitive task performance have proven useful in predicting rTMS treatment outcomes. Dresler et al. [39] published an exploratory, retrospective case study of patients with comorbid panic disorder and major depressive disorder who received high-frequency rTMS treatment. fNIRS measurements of the bilateral prefrontal cortex during an emotional Stroop task (with panic and neutral stimuli) before and after the course of TMS treatments showed that increased cortical activation bilaterally correlated with positive treatment responses. Deppermann et al. [40, 41] conducted a double-blind sham-controlled trial with 44 panic disorder patients using prefrontal cortex fNIRS measurements during a verbal fluency task and an emotional Stroop task at pre-treatment baseline and following a 4-week course of TMS therapy. Their findings confirmed that prefrontal hypoactivation during the tasks was corrected after TMS treatment, even when reported improvements in anxiety symptoms were not evident following the course of TMS. These methods were also used in another study by the same research group, which focused on spider phobias or arachnophobia [42]. However, in this study, TMS was not found to normalize functional dysconnectivity between the inferior frontal gyrus and DLPFC that was initially present in patients with spider phobias. The authors speculated that their results were confounded by the additional virtual reality challenge performed along with TMS in the study.

Finally, fNIRS has also been employed to assess whether cerebral oxygenation during self-regulatory control tasks could be modulated by TMS in a small cohort of patients with bulimia nervosa [43]. Significant decreases in hemoglobin concentrations in the left DLPFC were observed after a single TMS treatment delivered to the same region. This hemodynamic change correlated strongly with decreases in subjective ratings for desire to eat, urge to eat, and sense of hunger, thus providing preliminary data for the use of fNIRS for monitoring treatment response after sample exposure to TMS. The findings also suggest a novel causal mechanism for how TMS may exert inhibitory control and decrease food cravings in patients with eating disorders.

### Challenges and limitations

Despite the early successes of optical neuroimaging in TMS studies, there are a number of obstacles to overcome to advance the development of these imaging techniques. These include depth sensitivity, movement artifacts, and physiological-signal contamination.

The depth-sensitivity and penetration limits of NIR light are complex and multifaceted. These characteristics of NIR light depend on a plethora of NIRS application details, including the technology itself, the parameters used, and the neuroanatomical features of the subject being imaged. All these can significantly affect the physical properties of the light being absorbed and scattered by the brain, with differing depths possessing dynamic coefficient changes that require different calculations [23, 24]. The putative depth that can be reliably imaged (especially with commercial devices) is estimated to be in the 2–3 cm range (below the scalp surface) on average [45–47]. Further depths of 4–5 cm have been reported using higher wavelengths (808 nm or more) [48–50]. Due to its more advanced signal properties and sophisticated software interface, DOT is able to more consistently produce images in the 4–5 cm depth range, compared to fNIRS [25, 38, 51]. Additionally, recent experiments using time- or frequency-domain detection modes appear to mitigate some of the depth sensitivity limitations encountered with the standard continuous-wave design [52]. However, this problem certainly limits the utility of NIR light for imaging deeper structures within the brain, which can be salient for understanding many psychiatric disorders. For example, fMRI data collected before and after a 4- to 6- week course of TMS for depression have revealed that stimulation of the DLPFC indirectly modulates subgenual anterior cingulate cortex hyperactivity and normalizes dysregulated activity in the insula, amygdala, and other subcortical structures [10, 53, 54]. The ability to obtain functional images of these deeper areas during TMS is vital for understanding the effects of this treatment modality in patients with depression. Thus, depth sensitivity remains a barrier for optical neuroimaging.

Another common limitation inherent to any functional neuroimaging method is its vulnerability to movement artifacts. The changes in blood oxygenation and blood flow related to neural activity may be subtle relative to motion-related signal changes, thus creating challenges for the interpretation of hemodynamic responses in a specified region [55]. Such artifacts can degrade image quality, leading to confounded statistical analyses and inaccurate conclusions [56]. During optical neuroimaging procedures, motion occurring between the optical fiber and scalp of the subject can negatively affect image acquisition and cause decoupling and variation in the measured optical signal [57]. The most common type of motion artifact is a transient, high amplitude change in intensity, which subsides immediately after the motion ends. Depending on the magnitude of motion during the data acquisition procedures, whole datasets may be rendered useless when this type of artifact occurs in fMRI. However, when imaging with fNIRS or DOT, temporary and unaffected periods of data can still be captured and well utilized for analysis because of the higher tolerance of head/scalp motion secondary to design differences of the interface itself [58]. This tolerance is likely due to algorithm differences and the use of mesh-based headgear that can be fitted to the subject’s head with customized optode-scalp distances in optical imaging techniques, compared to the fixed and rigid design of fMRI. Standard techniques for preventing excessive movement artifacts include the careful design of optode arrays, minimization of subject motion using visual fixation, reduced stimuli, comfortable positioning, and the application of advanced post-processing methodologies [25]. Dynamic head motion can also be a problem for optical imaging during standard TMS treatments. Current solutions involve the use of rigid head fixation and chin resting frames, but the development of new algorithms to compensate for head motion during stimulation is needed to advance this area of clinical research [59, 60].

The threat of physiological signal contamination is common to all the imaging systems. Conventional functional imaging interfaces must account for signals propagated by non-neuronal sources, including proximal muscle activity, the subject’s cardiac and respiration cycles, spontaneous brain pulsations, and thermal noise related to the scanning process [58, 61, 62]. fNIRS and DOT must also mitigate the effects of environmental light pollution [23]. As such, the optimal imaging environment is made as dark as possible, which can be uncomfortable for some subjects. For TMS applications, in particular, the stimulation itself is accompanied by a vibration of the coil and a relatively loud clicking sound. Although these events are typically brief and associated with minimal motion, they can still introduce mechanical noise that should be accounted for in the selection of optode arrangements and data processing methodologies. Moreover, magnetic pulses emanating from the TMS coil can induce direct changes in the musculature surrounding the stimulation site and within the microvasculature of the scalp. New techniques involving multivariate superficial signal regressions have been developed to attempt to isolate neural signals from any surrounding confounders [63, 64]. Relative to other brain imaging methods, DOT also suffers from this problem less because of its inherent imaging reconstruction algorithm that separates the scalp layers from the brain [38, 64]. Other proposed approaches for minimizing these obstacles include positioning the TMS coil further from the source detectors (i.e., measuring the signal at a location more distant from the coil), designing a custom interface with carefully separated optodes, or placing the coil above the optical interface and increasing the baseline power to overcome the weakened and more distant resultant magnetic field [65]. Table 2 summarizes the differences between DOT, fNIRS, and other conventional functional neuroimaging techniques.

**Table 2:** Comparison of optical imaging to conventional functional imaging techniques

Techniques	Spatial resolution	Temporal resolution	Size/portability	Average cost (estimated per United States dollar)
fNIRS	Centimeter	Milliseconds	Small; portable	Thousands to tens of thousands
DOT	Millimeter	Milliseconds	Small; portable	Thousands to tens of thousands
fMRI	Millimeter	Seconds	Large; not portable	Millions
EEG	Centimeter	Milliseconds	Small; portable	Thousands to tens of thousands
MEG	Centimeter	Milliseconds	Large; not portable	Millions
PET	Millimeter	Minutes	Large; not portable	Millions
SPECT	Centimeter	Minutes	Large; not portable	Hundreds of thousands

### Future directions

Understanding the mechanisms underlying TMS effects and optimizing TMS protocols for improved treatment outcomes are critical areas of clinical research that can benefit from further experiments conducted with optical imaging techniques. The fNIRS and DOT studies published thus far have demonstrated preliminary but promising findings that will inform subsequent clinical trials and cross-validation studies. While many brain imaging modalities are useful for understanding the chronic effects of stimulation in both healthy volunteers and clinical samples, optical neuroimaging is particularly well-suited to advance the field in the optimization of TMS treatment features.

With regard to the selection of specific TMS parameters (e.g., pulse frequency, pattern, stimulation train durations, and rest intervals), fNIRS and DOT interfaces are uniquely poised to assist with the personalization of treatment protocols for psychiatric disorders. An ideal tool for guiding TMS protocol selection would permit each patient to briefly sample various stimulation parameters and rapidly generate target engagement metrics to inform clinicians of the neuromodulatory effects of each protocol. The results of this procedure could be used to estimate future symptom reduction following multiple applications of a specific type of stimulation. Given their localization capabilities and the real-time quantification of image analysis, optical modalities are perfectly suited for capturing such data, even in a naturalistic treatment setting. In addition, Shinba et al. [36] and Jiang et al. [38] demonstrated the feasibility of this paradigm, as immediate changes in hemoglobin concentrations during stimulation trains, as well as evoked changes observed during a single TMS treatment session, were reliably obtained and correlated with TMS response. Monitoring hemodynamic biomarkers such as those elicited by DOT during a typical 6-week course of daily TMS treatment sessions could theoretically inform the need for adjustments to various stimulation parameters in order to personalize the TMS protocol and optimize the effects of stimulation for the reduction of specific symptom clusters.

Another area to be explored in future research is the role of optical neuroimaging in generating data for functional connectivity analyses to guide the spatial targeting of TMS systems. For several psychiatric disorders (e.g., depressive disorders, anxiety disorders, schizophrenia), resting-state fMRI has been used to identify regions or networks within various cortical and subcortical structures, where dysfunctional connectivity appears to be associated with prominent symptoms of the disorder. As these pathological circuits are further elucidated, the use of neuroimaging-based navigation algorithms for precise placement of the TMS coil to target them may be critical for optimizing treatment outcomes [66–68]. Such a targeting strategy could be readily implemented with optical neuroimaging devices, given the sophisticated software interfaces and high degree of portability and flexibility they offer. fNIRS and DOT have been utilized in functional connectivity studies to identify brain networks in the visual, sensorimotor, language, and auditory systems in healthy subjects and patients with Alzheimer’s disease [69–72]. Further advancement of functional connectivity and other imaging-based TMS targeting approaches for treating neuropsychiatric disorders is imminent and could be accelerated by the deployment of optical imaging systems in research and clinical settings.

Finally, further development of optical imaging biomarkers is essential for implementing a personalized medical approach for noninvasive brain stimulation. The vast majority of optical neuroimaging studies on neuropsychiatric disorders have relied on absorption-based techniques for measuring hemoglobin as the main chromophore of interest. However, other chromophores may be viable candidates for the assessment of functional activity within the human brain. Cytochrome c oxidase (COX) is the final enzyme in the electron transport chain of the mitochondria. It is responsible for the maintenance of the transmembrane proton gradient, which is paramount for the synthesis of adenosine triphosphate, the main source of cellular energy. Mitochondrial dysfunction driven by impaired COX levels has been proposed as a contributing factor to the pathophysiology of depression and bipolar disorders [73]. A recent study by Holper et al. [74] used fNIRS for the first time to evaluate the COX chromophore and observed that lower COX levels correlated inversely with depression severity. In light of their potential for simultaneously acquiring functional data regarding the acute hemodynamic response as well as metabolic rates, optical imaging techniques may prove uniquely valuable for the advancement of our understanding of the biological underpinnings of psychopathology and for optimizing TMS treatment strategies.

## Conclusions

Optical neuroimaging techniques are highly promising tools for the study of TMS mechanisms and for the development of ways to optimize TMS therapeutic effects on psychiatric disorders. In this context, they stand out from other imaging methods owing to their combination of high resolution, portability, real-time capability, and relatively low cost. At present, there are no other well-developed or practical methods for accurately measuring functional brain activity with high spatial and temporal resolutions during the active application of TMS. There is a growing body of preliminary research describing the successful use of optical neuroimaging methods to predict TMS outcomes and elucidate the mechanisms relevant to psychiatric disease and recovery. However, much work remains to improve our noninvasive neuromodulation treatments and alleviate the burdens associated with these neuropsychiatric disorders. Through further investment in the development of these tools, fNIRS and DOT may play an essential role in advancing the science needed to understand salient neurocircuit pathologies and personalize TMS care for more rapid, robust, and durable treatment outcomes.

## Data Availability

Not applicable.

## Abbreviations

TMS: Transcranial magnetic stimulation
rTMS: Repetitive transcranial magnetic stimulation
fNIRS: Functional near-infrared spectroscopy
DOT: Diffuse optical tomography
MRI: Magnetic resonance imaging
CT: Computed tomography
fMRI: Functional magnetic resonance imaging
SPECT: Single photon emission computed tomography
PET: Positron emission tomography
MEG: Magnetoencephalography
EEG: Electroencephalography
DLPFC: Dorsolateral prefrontal cortex
NIR: Near-infrared
COX: Cytochrome c oxidase
HbT: Hemoglobin concentration
RMT: Resting motor threshold
HbO: Oxyhemoglobin concentration
HAM-D: Hamilton Depression Rating Scale
